# Quercitrin, the Main Compound in *Wikstroemia indica*, Mitigates Skin Lesions in a Mouse Model of 2,4-Dinitrochlorobenzene-Induced Contact Hypersensitivity

**DOI:** 10.1155/2020/4307161

**Published:** 2020-07-09

**Authors:** Jonghwan Jegal, No-June Park, So-Yeon Lee, Beom-Geun Jo, Sim-Kyu Bong, Su-Nam Kim, Min Hye Yang

**Affiliations:** ^1^College of Pharmacy, Pusan National University, Busan 46241, Republic of Korea; ^2^Natural Products Research Institute, Korea Institute of Science and Technology, Gangneung 25451, Republic of Korea

## Abstract

Hapten-induced contact hypersensitivity (CHS) is widely utilized to induce immune activation in animal models of allergic contact dermatitis. Our previous findings suggested that the 95% EtOH extract of *Wikstroemia indica* (L.) C. A. Mey. has antiallergic and anti-inflammatory effects in DNCB-treated CHS SKH-1 hairless mice. The aim of this study was to evaluate the protective effects of compounds isolated from the EtOAc fraction of *W. indica* in RBL-2H3 cells and 2,4-dinitrochlorobenzene- (DNCB-) induced CHS mice. Of eight compounds in *W. indica*, that is, umbelliferone, daphnoretin, wikstrocoumarin, (+)-syringaresinol, tricin, (+)-lariciresinol, *erythro*-guaiacylglycerol-*β*-coniferyl ether, and quercitrin, quercitrin exhibited the most antiallergic activity against antigen-induced *β*-hexosaminidase release and IL-4 mRNA expression, which are markers of degranulation in RBL-2H3 cells. After a 7-sensitizing period, 14 days of DNCB treatment with or without topical pimecrolimus (1%) or quercitrin (0.5%) treatment, quercitrin was found to suppress DNCB-induced increases in serum IL-4 and IgE concentrations and transepidermal water loss. These results indicate that quercitrin has therapeutic potential for treatment of allergies and allergy-related contact dermatitis.

## 1. Introduction

Contact hypersensitivity (CHS) is one of the most common skin inflammatory diseases and affects 15–20% of individuals worldwide [1]. Contact hypersensitivity is a type IV delayed hypersensitivity mediated by T cells and manifests as an eczematous skin condition that occurs when a substance (e.g., urushiol from poison ivy, a fragrance, or nickel) that acts as an allergen or hapten comes into contact with skin [2–4]. Contact hypersensitivity occurs in two phases, namely, a sensitization phase and an elicitation phase [5]. The sensitization phase occurs when a hapten applied to skin is first introduced to the immune system [6, 7]. Langerhans cells recognize haptens and then mature, migrate to lymph nodes, and present processed haptens to naive T cells. These sensitized naive T cells then migrate to *epidermis* and differentiate into effector T cells [4, 8]. These processes involve cytokines such as IL-1*β*, IL-12, and TNF-*α* [4, 5]. Subsequently, when sensitized skin is reexposed to a hapten, the elicitation phase of CHS begins. This reexposure of hapten to “sensitized” T cells results in the explosive differentiation and proliferation of T cells [9], and cytokines secreted by these T cells stimulate cells in skin, which leads to the recruitment of T cells and the enhancement of inflammation at hapten-challenged sites [9, 10]. The cytokines of INF-*γ*, IL-4, and IL-17 play major roles during the elicitation phase [5, 6].

Flavonoids are a group of aromatic ring compounds with a C6-C3-C6 composition and occur widely in plants (about 400 plant species have been identified) [11]. Flavonoids act as pigments that color fruits and flowers and are also involved in plant growth and defense [12]. Fruits, vegetables, and medicinal plants contain large quantities of flavonoids, and much research has shown they have many beneficial effects [13, 14]. In particular, several studies have demonstrated the antiallergic, anti-inflammatory, and antioxidant effects of flavonoids [15, 16]. Naturally occurring flavonoids reduce the productions of inflammatory cytokines secreted by mast cells, basophils, and Th cells in immunoglobulin *E-* (IgE-) mediated allergies [17–21]. *Wikstroemia indica* (L.) C. A. Mey. (Thymelaeaceae) is a plant native to Southeast Asia and China and is used in Chinese traditional medicine to treat coughs, syphilis, and arthritis [22]. *W. indica* contains diverse flavonoids, such as quercetin and epicatechin, with anti-inflammatory and antioxidant activities [23–25]. In a previous report, our findings supported the use of *W. indica* to treat types of allergic contact dermatitis like atopic dermatitis [26]. However, relatively few studies have indicated that flavonoids isolated from this plant are effective on skin inflammatory diseases. The aim of the present study was to isolate bioactive flavonoids from *W. indica* and to investigate their preventive and therapeutic potentials on CHS.

## 2. Materials and Methods

### 2.1. General Methods

NMR spectra were recorded using a Varian Unity INOVA 400 spectrometer (400 MHz for 1H and 101 MHz for 13C-NMR) (Agilent Technologies, Santa Clara, CA, USA) and Bruker Avance NEO 500 spectrometer system (COSY, HMQC, HMBC, and NOESY) (Bruker, Billerica, MA, USA). High-performance liquid chromatography (HPLC) was performed using a Shimadzu system (Shimadzu Corporation, Kyoto, Japan), which consisted of a LC-20AT pump, SPD-20A UV detector, and CBM-20A system controller. High-resolution electrospray ionization mass spectra (HRESIMS) were obtained using an Agilent 6530 Accurate-Mass Q-TOF LC/MS spectrometer system (Agilent Technologies, Santa Clara, CA, USA). Column chromatography (CC) was performed on silica gel (230–400 mesh, Merck, Germany) and Sephadex LH-20 (25–100 mM mesh, Pharmacia, Sweden) and thin-layer chromatography (TLC) on precoated silica gel 60 F254 (1.05554.0001, Merck, Germany) plates. Spots were visualized by spraying p-anisaldehyde solution.

### 2.2. Plant Material and Extraction

The aerial portions of *Wikstroemia indica* (L.) C. A. Mey (Thymelaeaceae) were collected from a village called Jobra in Chittagong, Bangladesh. A voucher specimen (PNU-0026) was deposited at the Medicinal Herb Garden of Pusan National University (Busan, Republic of Korea). Plant materials were authenticated by Dr. Sang Woo Lee (Korea Research Institute of Bioscience and Biotechnology). Dried *W. indica* (4 kg) was sonicated in 95% EtOH (12 L × 3) for 90 min and allowed to stand overnight. The extract solvent was then filtered through Advantec No. 2 filter paper (Advantec, Toyo Roshi Kaish, Ltd., Tokyo). The 95% EtOH extract of *W. indica* (247.6 g) was then obtained by evaporating the EtOH extract. The 95% EtOH extract of *W. indica* (WIE) was suspended in water (2.2 L) and fractioned versus *n*-Hex (15.7 g), EtOAc (20.1 g), and *n*-BuOH (16.4 g).

### 2.3. Isolation of Compounds from *Wikstroemia indica*

WIE (247.6 g) was suspended in distilled water (2.2 L) and sequentially partitioned versus *n*-Hex (4.6 L), EtOAc (7.2 L), and *n*-BuOH (6.4 L). In addition, the EtOAc fraction of *W. indica* 95% EtOH extract (WIE-EtOAc, 20.1 g) was chromatographed on a silica gel column using an *n*-hexane-EtOAc (10 : 1 ⟶ 100% MeOH, gradient system) to yield twelve fractions (WIE1–WIE12). Compound 1 (umbelliferone, 117.8 mg) was obtained as pure powder by filtering fraction WIE4. Compound 2 (daphnoretin, 16.5 mg) was obtained by recrystallizing fraction WIE7 (1.06 g) from MeOH and refractioning to produce seven subfractions (WIE7-1–WIE7-7) by silica gel CC (column chromatography) using a CH_2_CI_2_-MeOH (7 : 1 ⟶ 100% MeOH) gradient system. Subfraction WIE7-1 (128.6 mg) was fractionated into six subfractions (WIE7-1-1–WIE7-1-6) by Sephadex LH-20 CC using 100% MeOH as eluant. Compound 3 (wikstrocoumarin, 2.4 mg) was obtained by filtering fraction WIE7-1-3. Subfraction WIE7-1-2 (17.4 mg) was fractionated into five subfractions (WIE7-1-2-1–WIE7-1-2-5) by PTLC (preparative thin-layer chromatography) using CH_2_CI_2_-MeOH (15 : 1) as eluant. Subfraction WIE7-1-2-5 (6.2 mg, Rf 0.88) was subjected to RP HPLC (Watchers 120 ODS-BP, S-10 *µ*m, 250 × 10 mm; detection, UV at 254 nm; flow rate, 2 mL/min) and eluted with an isocratic ACN-H_2_O system (3 : 7, 40 min) to yield compound 4 ((+)-syringaresinol, 1.4 mg, *t*_R_ 27 min). Subfraction WIE7-2 (158 mg) was fractionated into seven subfractions (WIE7-2-1–WIE7-2-7) by Sephadex LH-20 CC using 100% MeOH as eluant. Compound 5 (tricin, 1.6 mg) was obtained as pure powder by filtering fraction WIE7-2-7. Subfraction WIE7-2-3 (40.2 mg) was subjected to PTLC using CH_2_Cl_2_-MeOH (10 : 1) as eluent to yield compound 6 ((+)-lariciresinol, 8 mg, R*f* 0.64). Fraction WIE8 (441.1 mg) was subjected to silica gel CC using a CH2Cl2-MeOH (30 : 1 ⟶ 100% MeOH, gradient system) to yield five fractions (WIE8-1–WIE8-5). Subfraction WIE8-3 (61.7 mg) was fractionated into five subfractions (WIE8-3-1–WIE8-3-5) by Sephadex LH-20 CC using 100% MeOH as eluent, and subfraction WIE8-3-2 (10.3 mg) was subjected to RP HPLC (Watchers 120 ODS-BP, S-10 *µ*m, 250 × 10 mm; detection, UV at 254 nm; flow rate, 2 mL/min) and eluted with a MeOH-H_2_O isocratic system (4 : 6, 30 min) to yield compound 7 (erythro-guaiacylglycerol-*β*-coniferyl ether, 1.9 mg, *t*_R_ 19 min). Fraction WIE9 (10.5 g) was chromatographed on a silica gel column using CH_2_Cl_2_-MeOH (10 : 1 ⟶ 100% MeOH, gradient system) as eluent to yield eight fractions (WIE9-1–WIE9-8). Subfraction WIE9-7 (275.2 mg) was fractionated into four subfractions (WIE9-7-1–WIE9-7-4) by Sephadex LH-20 C.C using 100% MeOH as eluent. Compound 8 (quercitrin, 101.4 mg, purity ≥ 98%, HPLC) was obtained as pure powder by filtering fraction WIE9-7-3.

### 2.4. Cell Culture

The RBL-2H3 mast cell line was purchased from the American Type Culture Collection (ATCC, #CRL-2256, Manassas, VA, USA) and cultured in minimum essential medium (MEM) supplemented with 2 mM·L-glutamine, 100 *μ*/mL penicillin, 100 *μ*g/mL streptomycin, and 10% v/v heat-inactivated fetal bovine serum (FBS). Cells were maintained at 37°C in a humidified atmosphere containing 5% CO_2_.

### 2.5. Analysis of IL-4 mRNA Expression by Real-Time PCR

To assess the effect of compounds isolated from *W. indica* on the mRNA expression of interleukin 4 (IL-4) in RBL-2H3 cells, cells were sensitized to DMSO or compounds (10 *μ*M and 30 *μ*M) for 30 minutes and later stimulated with phorbol 12-myristate 13-acetate (PMA, 50 ng/mL) and ionomycin (1 *μ*M) for 16 hours. Total RNA was extracted using RNAiso Reagent (TaKaRa, Shiga, Japan), and SYBR Green based quantitative real-time PCR (qPCR) was performed according to the manufacturer's instructions. Data were analyzed using 7500 System SDS software version v1.3.1 (Applied Biosystems). The primer sequences used for IL-4 were 5′-ACC TTG CTG TCA CCC TGT TC-3′ (forward) and 5′-TTG TGA GCG TGG ACTCAT TC-3′ (reverse), and the primer sequences of the housekeeping gene (*β*-actin) were 5′-TCA TCA CCA TCG GCA ACG-3′ (forward) and 5′-TTC CT GAT GTC CAC GTC GC-3′ (reverse).

### 2.6. Release Activity of *β*-Hexosaminidase

RBL-2H3 cells were suspended in MEM media at 1 × 10^5^ cells per well in a 24-well plate and sensitized with anti-DNP IgE for 24 hours. They were then washed in Siraganian buffer (pH 7.5; 100 ng/ml DNP-IgE, 119 mM NaCl, 5 mM KCl, 0.4 mM MgCl_2_, 25 mM PIPES, and 40 mM NaOH) twice and incubated for 10 minutes in the same buffer. Cells were then treated with DMSO or compounds (10 *μ*M and 30 *μ*M) for 1 hour, with 1 *μ*g/mL DNP-BSA antigen for 30 minutes to induce cell degranulation, and then supernatants obtained by microcentrifugation were transferred to 96-well plates and incubated with substrate solution (1 mM poly-N-acetyl glucosamine in 0.1 M sodium citrate buffer) for 3 hours at 37°C. Finally, stop solution was added and well absorbances were measured using a microplate reader at 405 nm.

### 2.7. Animal Experiments

SKH-1 hairless mice (females, 6 weeks old) were procured from Orient Bio Inc. (Seongnam, Republic of Korea). All mice were housed under 12 h light-dark cycle and controlled conditions (RH 55 ± 5% and 25 ± 5°C) for 1 week before experimentation and fed a standard laboratory diet and water *ad libitum*. Mice care and experimental protocols were approved by the Institutional Animal Care and Use Committee of KIST (Certification No. KIST-2016-011) beforehand and were in accordance with the Guide for the Care and Use of Laboratory Animals published by the US National Institute of Health (NIH Publication No. 85–23, 2011 revision). For CHS induction, SKH-1 hairless mice were acclimated for 1 week and then divided into 4 groups of 7 animals; a normal control group (animals were treated with distilled water), a negative control group (0.1% DNCB plus vehicle), a positive control group (0.1% DNCB and 1% pimecrolimus), and an experimental group (0.1% DNCB sensitized plus treatment with 1% quercitrin). On day 1, mice were sensitized by applying 1% DNCB (100 *μ*L) dissolved in propylene glycol and ethanol (7 : 3) topically to dorsal skins for 7 days to induced CHS (day 1 to day 7). Mice were stimulated with 0.1% DNCB (100 *μ*L) for 2 weeks (day 8 to 21) at 2-day intervals, and DNCB + quercitrin group and DNCB + pimecrolimus (Elidel^®^) group were treated with 0.5% quercitrin (100 *μ*L) and 1% pimecrolimus cream twice daily (day 8 to 21). On day 22, animals were sacrificed, and dorsal skin tissue was removed for histological examination and blood was collected from abdominal aortas for serum IgE and IL-4 testing.

### 2.8. Measurement of Skin Conditions

All experiments used to assess skin conditions were performed at 25 ± 5°C and 50 ± 5% RH. Transepidermal water loss (TEWL) was measured using a Tewameter TM210 (Courage and Khazaka, Cologne, Germany) with an open chamber system. Skin barrier damage was expressed in g/m^2^/h. A SKIN-O-MAT device (Cosmomed, Ruhr, Germany) was used to measure hydration levels of mouse skins.

### 2.9. Total Serum IgE and IL-4 Levels

Blood was collected from abdominal aortas on day 22, centrifuged at 10,000 rpm for 15 minutes at 4°C, and stored at −70°C until needed for IgE and IL-4 measurements. Total serum IgE and IL-4 levels were quantified using an enzyme-linked immunosorbent assay kit (eBioscience, San Diego).

### 2.10. Histological Analysis

Dorsal skin samples were fixed in 10% neutral buffered formalin (NBF) and embedded in paraffin. Sections of dorsal skin (4–6 *μ*m thickness) were stained with hematoxylin and eosin (H&E) to evaluate eosinophil infiltration or with toluidine blue to count mast cell numbers. Dorsal skin tissues were visualized under a light microscope (Olympus CX31/BX51, Olympus Optical Co., Tokyo) and photographed (TE-2000U, Nikon Instruments Inc., Melville, USA).

### 2.11. Statistical Analysis

Statistical analysis was performed using one-way ANOVA followed by Tukey's HSD multiple comparison test. Results are presented as mean ± standard deviations, and statistical significance was accepted for *p* values < 0.05.

## 3. Results

### 3.1. Isolation of Compounds from WIE-EtOAc and Their Effects on *β*-Hexosaminidase Release from and IL-4 Expression in RBL-2H3 Cells

**Figure 1:**
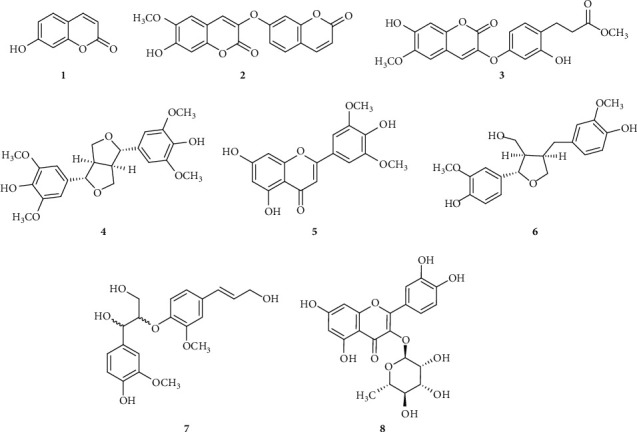
Chemical structures of compounds isolated from EtOAc fraction of the 95% EtOH extract of *W. indica* (WIE-EtOAc): 1, umbelliferone; 2, daphnoretin; 3, wikstrocoumarin; 4, (+)-syringaresinol; 5, tricin; 6, (+)-lariciresinol; 7, *erythro*-guaiacylglycerol-*β*-coniferyl ether; 8, quercitrin.

The *in vitro* antiallergic and anti-inflammatory activities of WIE and its fractions (*n*-hexane, EtOAc, *n*-BuOH, and water) were evaluated and the EtOAc fraction was the most active in RBL-2H3 cells (data not shown). The WIE-EtOAc was subjected to silica gel column chromatography, Sephadex LH-20 column chromatography, and RP HPLC to yield eight known compounds, namely, umbelliferone (1), daphnoretin (2), wikstrocoumarin (3), (+)-syringaresinol (4), tricin (5), (+)-lariciresinol (6), *erythro*-guaiacylglycerol-*β*-coniferyl ether (7), and quercitrin (8) (Figure 1). Compounds were identified by comparing ^1^H-NMR, ^13^C-NMR, and MS data with literature values.

**Figure 2:**
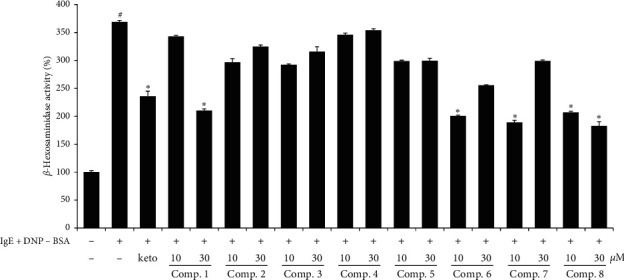
Effects of compounds isolated from WIE-EtOAc on *β*-hexosaminidase release from IgE-activated RBL-2H3 cells. Results are presented as mean ± SDs of two independent experiments performed in triplicate. ^#^*p* < 0.05 versus vehicle controls; Ket: 35 *μ*M ketotifen.

**Figure 3:**
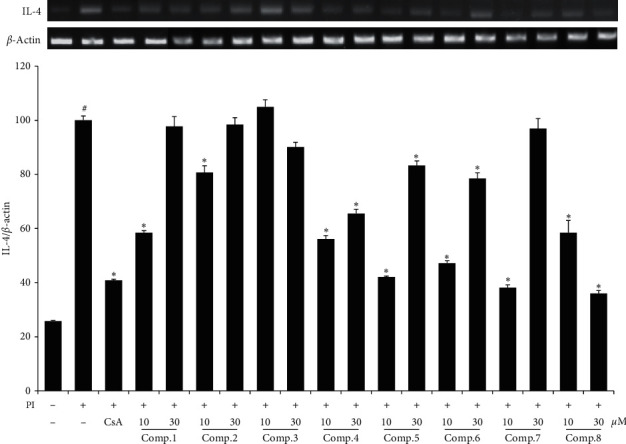
Effects of compounds isolated from WIE-EtOAc on IL-4 mRNA expression in PI-activated RBL-2H3 cells. Results are presented as mean ± SDs of two independent experiments performed in triplicate. ^#^*p* < 0.05 versus vehicle controls; ^*∗*^*p* < 0.05 versus PI. CsA: 1 *μ*M cyclosporine A.

To investigate the inhibitory effect of WIE-EtOAc on cell degranulation, RBL-2H3 cells were sensitized with IgE and activated with DNP-BSA. The results obtained showed that degranulation increased 3 times more in DNP/IgE-induced cells than in controls. However, treatment with compounds 1, 6, 7, and 8 effectively suppressed antigen-mediated *β*-hexosaminidase release from cells (Figure 2). The suppressive effects of compounds in WIE-EtOAc on IL-4 mRNA expression were investigated using phorbol 12‐myristate 13‐acetate (PMA) + ionomycin- (PI-) stimulated RBL-2H3 cells. It was observed that IL-4 mRNA expression in PI treated cells was greater than in controls, but that compounds 1, 2, 4, 5, 6, 7, and 8 reduced IL-4 mRNA levels (Figure 3). In particular, quercitrin (8) was the compound that suppressed DNP/IgE-induced *β*-hexosaminidase release (74.1% inhibition) and IL-4 mRNA expression by PI (86.7% inhibition) at 30 *μ*M.

### 3.2. Effects of Quercitrin from WIE-EtOAc on DNCB-Induced CHS in SKH-1 Hairless Mice

**Figure 4:**
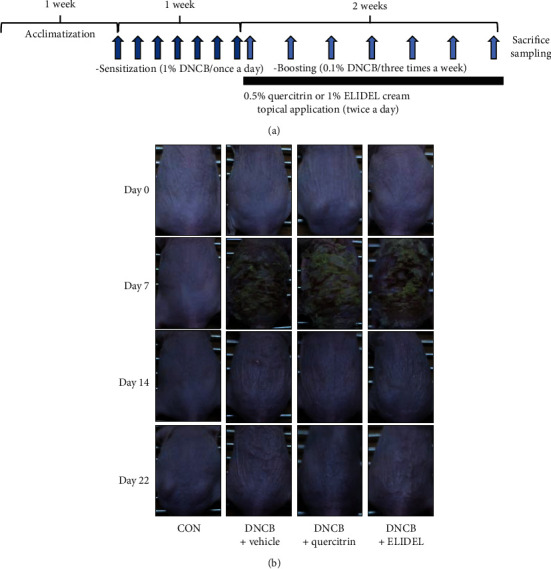
Effects of quercitrin on the development of DNCB-induced CHS in SKH-1 hairless mice. (a) Schematic representation of the experiment. (b) Clinical features of DNCB-induced skin symptoms. Values represent the mean SD (*n* = 7). CON: vehicle control group; DNCB + vehicle: DNCB-treated control group; DNCB + quercitrin: DNCB and 0.5% quercitrin-treated group; DNCB + Elidel: DNCB and 1% Elidel-treated group.

To investigate the effects of quercitrin from WIE-EtOAc on DNCB-induced skin symptoms, dermatitis levels were evaluated using skin images. The procedure used to establish the DNCB-induced CHS model is shown in Figure 4(a). DNCB treatment for 3 weeks resulted in severe CHS-like skin symptoms, that is, dried skin, cornification, exudation, and erythema, and these symptoms were markedly improved by 0.5% quercitrin treatment for 2 weeks and this improvement was similar to that observed in the 1% Elidel-treated group (the positive control group) (Figure 4(b)).

### 3.3. Effects of Quercitrin from WIE-EtOAc on Skin Histopathological Changes in DNCB-Induced CHS Mice

**Figure 5:**
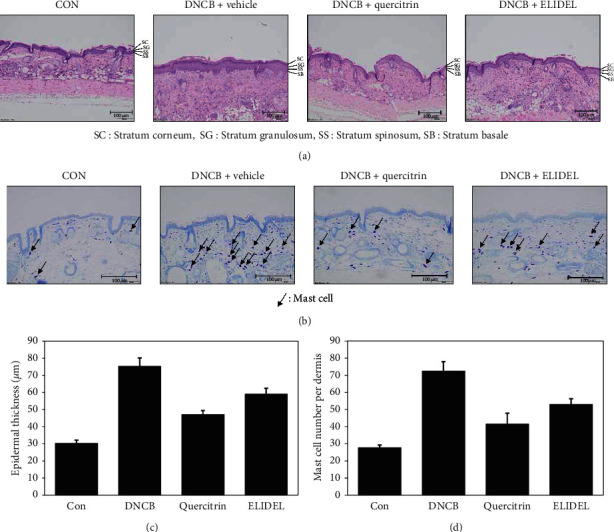
Effects of quercitrin on skin histopathological changes, epidermal thickness, and mast cell numbers in DNCB-induced CHS mice. (a) H&E staining. (b) Toluidine blue staining. (c) Epidermal thicknesses. (d) Mast cell numbers. Values represent the mean SD (*n* = 7). CON: vehicle control group; DNCB + vehicle: DNCB-treated control group; DNCB + quercitrin: DNCB and 0.5% quercitrin-treated group; DNCB + Elidel: DNCB and 1% Elidel-treated group. ^#^*p* < 0.05 versus the CON group; ^*∗*^*p* < 0.05 versus the DNCB group.

To evaluate the histological changes induced by quercitrin in the dorsal skins of DNCB-induced CHS mice, we used H&E and toluidine blue staining. H&E results showed that *epidermis* thickness was significantly greater in the DNCB group than in the CON group. However, *epidermis* thickness in the 0.5% quercitrin group was 77.8% lower than that in the DNCB group (Figures 5(a) and 5(c)). Toluidine blue staining showed mast cell numbers were greater in the DNCB group than in the CON group and that mast cell numbers were 66.7% lower in the quercitrin group (Figures 5(b) and 5(d)), which was a greater reduction in numbers than that observed in the Elidel group.

### 3.4. Effects of Quercitrin from WIE-EtOAc on Serum IgE and IL-4 Levels in DNCB-Induced CHS Mice

**Figure 6:**
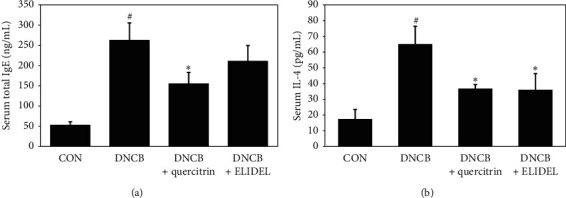
Effects of quercitrin on serum IgE and IL-4 levels in DNCB-induced CHS mice. (a) Serum total IgE levels. (b) Serum IL-4 levels. ^#^*p* < 0.05 versus the CON group; ^*∗*^*p* < 0.05 versus the DNCB group.

To measure levels of IgE and IL-4, blood samples were collected from abdominal aortas of DNCB-induced SKH-1 hairless mice and serum IgE and IL-4 concentrations were measured by ELISA. IgE was 5.2-fold higher and IL-4 was 3.8-fold higher in the DNCB group than in the CON group. However, 0.5% quercitrin treatment significantly decreased the serum IgE and IL-4 level increases induced by DNCB to 52.4% and 62.5%, respectively (Figure 6).

### 3.5. Effects of Quercitrin from WIE-EtOAc on Skin Barrier Function in DNCB-Induced CHS Mice

**Figure 7:**
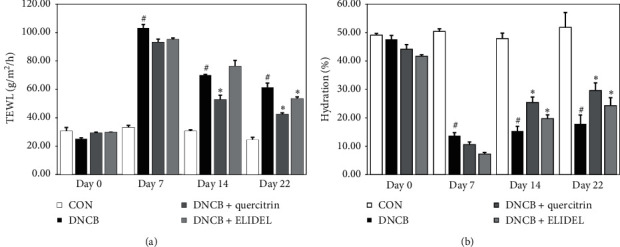
Effects of quercitrin on skin barrier function in DNCB-induced CHS mice. (a) Transepidermal water loss (TEWL). (b) Skin hydration. ^#^*p* < 0.05 versus the CON group; ^*∗*^*p* < 0.05 versus the DNCB group.

Changes in skin barrier function after treatment with 0.5% quercitrin for 2 weeks were evaluated by measuring TEWL and skin hydration. The results showed that TEWL was 2.7-fold higher and skin hydration was 0.3-fold lower in the DNCB group than in the CON group. However, 0.5% quercitrin treatment significantly reduced this TEWL DNCB-induced increase to 50% and improved skin hydration to 32.4% (Figure 7).

## 4. Discussion

The skin is made up of several tightly connected layers that are in combination function as a primary barrier, which protects the body from harm, prevents the entry of exogenous substances, and reduces water loss [27]. If the skin barrier is damaged, external antigens are likely to penetrate, and this causes T-helper 2 (Th2) cell-mediated immune response and subsequent inflammation [28]. Furthermore, excessive, prolonged inflammatory reactions cause tissue damage, which in turn detrimentally affects the skin barrier and establishes a vicious cycle [29]. Skin barrier dysfunction and immune system dysregulation are the main causes of skin inflammatory diseases [30]. Because skin barrier dysfunction plays an important role in the development of allergic contact dermatitis, strengthening this barrier is considered a primary strategy for preventing and treating contact dermatitis [31, 32]. Several methods can be used to assess skin barrier dysfunction, and TEWL provides a simple and noninvasive means of doing so [33]. The TEWL values of patients with allergic contact dermatitis are higher than those of healthy individuals [34].

Our previous study of WIE revealed that its topical application on the dorsal skins of mice decreased the severity of DNCB-induced allergic contact dermatitis by reducing edema, erythema, and inflammation [26]. The therapeutic effects of WIE against hapten-induced CHS are believed to be due to skin barrier recovery and the subsequent inhibition of IL-4. Accordingly, attempts have been made to develop a procedure for separating biologically active components from WIE. In the present study, flavonoids, lignans, and coumarins were isolated from an EtOAc fraction of WIE using various chromatographic methods, and of these isolated compounds, quercitrin (a glycoside of quercetin) exhibited strong *in vitro* inhibitory activities against DNP/IgE-induced *β*-hexosaminidase release and PI-induced IL-4 mRNA expression. *In vivo* testing using DNCB-induced CHS mouse model was performed to assess the therapeutic potential of quercitrin for the treatment of contact dermatitis. Murine CHS models are often used to investigate the pathogeneses of human and animal allergic CHS [1]. Typical haptens, such as 2-chloro-1,3,5-trinitrobenzene (TNCB), 2,4-dinitro-1-fluorobenzene (DNFB), 2,4‐dinitrochlorobenzene (DNCB), and oxazolone, are known to induce rapid CHS in animals [1, 35]. In the present study, the severe CHS reaction induced by DNCB was clearly suppressed by treatment with 0.5% quercitrin for 2 weeks. Furthermore, barrier functional damage caused by DNCB was largely prevented by quercitrin. According to the previous literature, Th2 cytokine IL‐4 enhances expression of kallikrein 7 (KLK7) and downregulates expression of filaggrin, a key epidermal barrier protein in allergic skin disease [36]. The inhibitory effects of quercitrin on IL-4 production may provide an important clue for the mechanism underlying its skin barrier protective effect.

RBL-2H3 cells are an excellent experimental model for studying the effects of drug involved in Th2 immune responses and have characteristics resembling those of mast cells, for example, they secrete cytokines and histamine, which induce IgE receptor expression and immune responses on cell surfaces [37, 38]. In the present study, the antiallergic and anti-inflammatory effects of compounds isolated from WIE were determined using procedures previously used in RBL-2H3 cells. Out of the eight compounds isolated, quercitrin most effectively suppressed *β*-hexosaminidase release and IL-4 mRNA expression in RBL-2H3 cells. This data suggests that quercitrin partially inhibits the differentiation of Th0 cells into Th1 cells and decreases IL-4 release in DNCB-stimulated mice. Cytokines produced by T cells, such as IL-4, IL-10, and IFN-*γ*, play keys roles during the pathogenesis of CHS [4]. In particular, IL-4 is a pleiotropic cytokine of Th2 cells that regulates immunoglobulin isotype switching to IgG4 and IgE [39]. In a previous study, it was suggested that total loss of endogenously produced IL-4 in BALB/c mice was closely associated with impairment of DNCB-induced CHS [35]. This result supports the importance of IL-4 as an immune response regulator during the early phase of skin CHS. We observed the productions and expressions of IL-4 and IgE were significantly increased in DNCB-sensitized mice in-line with CHS induction and that dermal application of quercitrin, a major constituent of *W. indica*, greatly reduced IL-4 expression, which suggested quercitrin suppresses Th2 immunity in mice CHS.

## 5. Conclusion

Our study shows that quercitrin isolated from *W. indica* has a novel protective effect against CHS in the DNCB-induced SKH-1 hairless mouse model. Furthermore, quercitrin significantly attenuated DNP/IgE-induced *β*-hexosaminidase release and PI-induced IL-4 mRNA expression in RBL-2H3 cells. The inhibitory effects of quercitrin on IL-4 and IgE in CHS mice suggest that it might be useful for treating allergic skin disorders. Furthermore, the observed improvement of skin barrier function by quercitrin would seem to have considerable pharmacological potential for the treatment of contact dermatitis.

## Data Availability

The datasets used and/or analyzed during the current study are available from the corresponding author upon reasonable request.

## Abbreviations

CHS: Contact hypersensitivity
DNCB: 2,4-Dinitrochlorobenzene
IgE: Immunoglobulin E
IL: Interleukin
WIE: Wikstroemia indica aerial parts 95% EtOH extract
Th2: T‐helper cell type‐2
TEWL: Transepidermal water loss.
